# Diffusion-localization transition caused by nonlinear transport on complex networks

**DOI:** 10.1038/s41598-018-23675-x

**Published:** 2018-04-03

**Authors:** Koutarou Tamura, Hideki Takayasu, Misako Takayasu

**Affiliations:** 1 Institute of Innovative Research, Tokyo Institute of Technology, 4259-S1-3, Nagatsuta-cho, Midori-ku Yokohama, 226-8503 Japan; 2 Sony Computer Science Laboratories, 3-14-13, Higashigotanda, Shinagawa-ku Tokyo, 141-0022 Japan

## Abstract

We analyzed nonlinear transport as defined for directed complex networks, where the flux from one node to a neighboring node is given preferentially according to the scalar quantities at the neighbor nodes. This is known as the generalized gravity interaction. In our research, we discovered a novel phase transition type. In the diffusion phase, the scalar quantity is scattered over the whole system, whereas in the localization phase, the flow tends to form localized confluence patterns owing to nonlinearity, resulting in the appearance of special nodes that irreversibly attract huge amounts of flow. We analytically considered the transition for selected network configurations, demonstrating that the transition point depends on the network topology. We also demonstrated that the diffusion phase of this transport model fits well with data from business firms, implying that the whole network structure can be used to model money flow in the real world.

## Introduction

Physicists have recently become interested in various social phenomena resulting from human activity that can be described in terms of transport systems, such as migration and traffic^1–7^, international and interfirm trade^8–13^, and telecommunication^14^. These transport systems in a social system universally exhibit a nonlinear interaction between actors that is called the gravity interaction. Transport phenomena caused by human activity are generally expected to be described as nonlinear interaction in a complex network. Unlike material transport, which is usually defined in three-dimensional Euclidean space, these social phenomena are often analyzed in the framework of complex networks as a first-step approximation of strongly heterogeneous interactions.

Transport phenomena on a given network structure are also important research topics from a physical viewpoint. The most basic transport process in complex networks is a simple random walk to a node chosen randomly among linked neighbor nodes with equal probability, which becomes equivalent to thermal diffusion when a small probability of spontaneous jumping to any node is added to avoid walkers becoming stuck^15–22^. Thermal diffusion in Euclidean space results in a uniform steady state; however, in complex networks, the steady-state distribution depends on the network structure. This idea has been applied to the basic technology of Google’s PageRank algorithm, in which a node is a homepage, and a link represents a connection by a click^15^.

The thermal diffusion model has been generalized to biased random walk models while the linearity of the process has been maintained; for example, the transport probability is given in proportion to a fractional power of the link number of the destination node^16,20,22^. In a biased random walk model that assumes a biased transport probability as a function of the network distance from the origin, a localization transition occurs; that is, a random walker never visits the origin in some parameter range^22^.

These types of diffusion-type generalized linear transport are being analyzed intensively for various complex networks^16–20,23^ and used for an elementary process in a social transport model^20^, although the gravity interaction is commonly observed and reported in many fields. On the basis of the bias of the flow, the authors proposed a method to extract the mainstream and basin structure of complex network flow and demonstrated a money flow case in a business transaction network^24^.

In this paper, we propose a nonlinear transport model based on a gravity interaction broadly observed in social phenomena. In the next section, we introduce a transport model of a scalar quantity based on the so-called gravity interaction in which the flow intensity on a link is proportional to the products of the scalar quantities at both nodes. In Sec. 3, we analyze this nonlinear-type transport on a two-dimensional (2D) lattice and find a phase transition of the diffusiveness. In Sec. 4, we show that the system exhibits a transition between the diffusion phase and the localization phase, where the transition point is determined by both the key control parameter, which is the ratio of the fractional powers of the exponents, and the underlying network structure. In Sec. 5, we apply our model to a real interfirm trading network to evaluate the transition point of the network. Finally, we summarize our discussion.

## Modeling nonlinear flow on social networks

**Figure 1:**
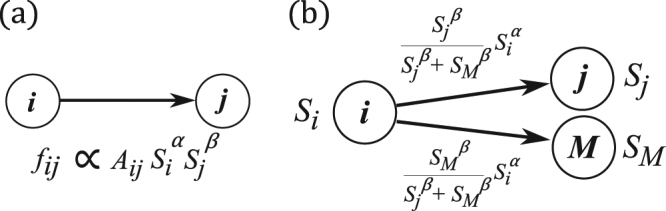
Schematic diagrams of nonlinear transport. (**a**) Interaction strength between a pair. (**b**) Share ratio.

The structure of a network is fully described by the adjacency matrix element *A*_*ij*_, which is equal to 1 if there is a directed link from the *i*-th node to the *j*-th node and equal to 0 otherwise. We consider transport of a conservative scalar quantity such as charge or current on this network. The charge on the *i*-th node at time *t* is denoted as *S*_*i*_(*t*), and we assume that the current from the *i*-th node to the *j*-th node, *f*_*ij*_(*t*), is proportional to the product of the fractional powers of the scalar quantities, *f*_*ij*_ ∝ *A*_*ij*_*S*_*i*_(*t*)^*α*^*S*_*j*_(*t*)^*β*^, as shown schematically in Fig. 1(a), where *α* and *β* are nonnegative constants.

When *β* = 0, the magnitude of the current does not depend on the charge of the destination, and the transport corresponds to a simple random walk or ordinary thermal diffusion. When *β* > 0, the current from a node depends on the charge of the destination, so if there are more than two destinations, the node with the highest charge receives more current than nodes with lower charge.

There are many empirical studies on estimation of the values of *α* and *β*. For example, both *α* and *β* are approximately 1.0 for human flow between two cities, where the populations are regarded as the charges^3–7^. Further, similar exponent values are estimated for money flow; in international trade, the charges represent the GDPs of each country^8,11,13,14^. In some cases, the exponents are asymmetric, for example, (*α*, *β*) = (0.46, 0.64)^4^ and (*α*, *β*) = (0.30, 0.64)^5^ in human flow, and it was concluded that the exponent of the destination is larger if the distance between the locations is less than a certain length. The authors have previously analyzed business firm transaction data compiled by Teikoku Databank, Ltd., and showed that the annual transaction volume from the *i*-th firm to the *j*-th firm is approximated by the generalized gravity interaction with the exponents *α* = 0.8~0.9, *β* = 0.3~0.5^9^.

Assuming generalized gravity interaction transport on each link of the directed network, as given in Fig. 1(b), and adding the effects of dissipation and injection for each node, we introduce the following set of dynamic equations.

1 $$\frac{d{S}_{M}^{\alpha }}{dt}\,\propto \,\sum _{i=1}^{N}{A}_{iM}\frac{{S}_{i}^{\alpha }{S}_{M}^{\beta }}{{\sum }_{j=1}^{N}{A}_{ij}{S}_{j}^{\beta }}-\,(1+{\nu }_{M}){S}_{M}^{\alpha }+{F}_{M}$$

Here *S*_*M*_ denotes the charge of the *M*-th node, *N* is the total number of nodes, and *ν*_*M*_ and *F*_*M*_ are the dissipation coefficient and injection, respectively, for the *M*-th node. In the left hand side of Eq. (1) we assumed a velocity term of state M for deriving a steady-state solution with a virtual parameter t which is not corresponding to the real time. When *β* = 0, the currents emitted from a node are equal for all neighbors, a phenomenon known as equipartition; on the other hand, when *β* = ∞, the largest neighbor receives the entire current, causing a monopoly.

We pay particular attention to the steady state of this system when *ν*_*M*_ and *F*_*M*_ are nonnegative constants that are independent of *M*. In this model the dissipation and injection effect cannot be determined directly by our data, which does not contain any information of the dissipation and injection of each firm. However, supposed that inflow and outflow of each firm are determined as a gravitational flow, the dissipation term and the injection coefficients are derived by asymptotic behavior comparing the balance between the total inflow and the total outflow of each firm because the currents between nodes are conserved in this transport.

To simplify the theoretical analysis, we can reduce the number of parameters in Eq. (1) by introducing the variable ${Q}_{i}={S}_{i}^{\alpha }/F$ and by setting the scale factor to 1.

2 $$\frac{d{Q}_{M}}{dt}=\sum _{i=1}^{N}{A}_{iM}\frac{{Q}_{i}{Q}_{M}^{\gamma }}{{\sum }_{j=1}^{N}{A}_{ij}{Q}_{j}^{\gamma }}-\,(1+\nu ){Q}_{M}+1$$

Here *γ* = *β*/*α* is the key parameter that controls the nonlinearity of the transport; namely, the parameters *α* and *β* contribute to the power exponent of the distribution of the steady-state solution, while the parameters *ν* and *F* determine the total amount of the flow: ∑_*M*_*Q*_*M*_ = *NF*/*ν*. For any given network structure, we consider the steady states of Eq. (2).

### Diffusiveness of nonlinear flow

**Figure 2:**
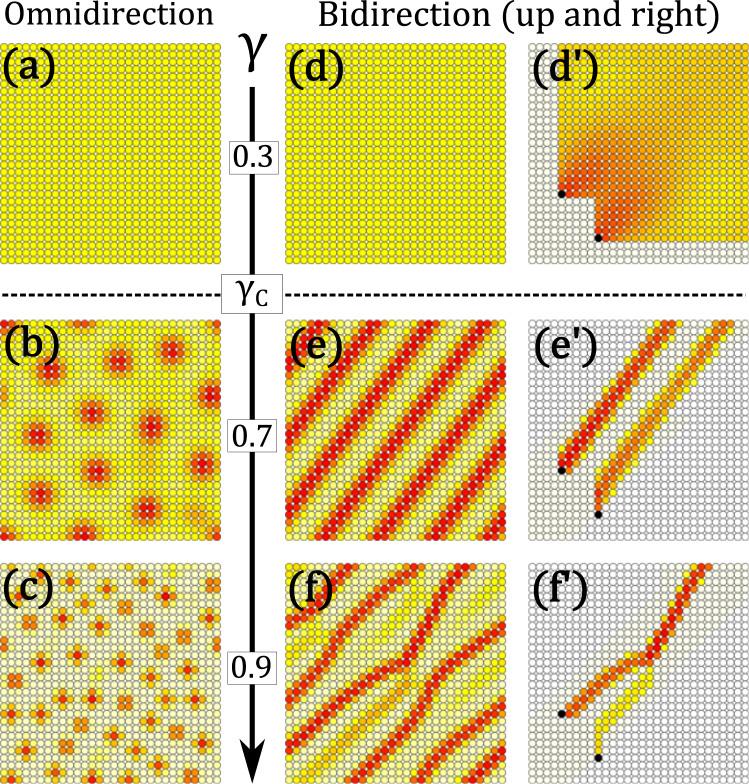
Steady-state patterns on a 2D periodic regular lattice (red: large, yellow: intermediate, gray: small, normalized in each figure). In (**a**)–(**c**), the currents between neighbors can flow in both directions. In (**d**)–(**f**), current can flow only upward and to the right. In (**d**′)–(**f**′), the flow of charges emitted from the black source points is traced. The numerical calculation was performed with *ν* = 0.01.

To clarify the role of nonlinear transport, we first consider Eq. (2) on a regular 2D periodic lattice. Figure 2(a)–(c) show examples of the steady states of Eq. (2) with *ν* = 0.01 for three typical values of *γ*, 0.3, 0.7, and 0.9, for an omnidirectionally connected node. Starting with a randomly assigned initial condition, as in the case where *γ* = 0.3, all nodes have the same charge in the steady state, implying that the steady solution is uniquely independent of the initial condition. However, if *γ* = 0.7 or 0.9, the steady state charges are nonuniform and localized, as shown in Fig. 2(b) and (c). We can confirm that the steady states depend on the initial condition, so there are multiple steady solutions.

We also consider a bidirectionally connected node at which the flow is limited to the upward and rightward directions. When *γ* = 0.3 [Fig. 2(d)], the steady-state solution is uniform, which is similar to the result for omnidirectional flow [Fig. 2(a)]. When *γ* = 0.7 or 0.9, as shown in Fig. 2(e) and (f), respectively, the steady-state charge patterns are represented by nonuniform stripes, and we can confirm that the pattern details depend on the initial condition.

To see the differences between Fig. 2(d)–(f) more clearly, we trace the flow of charges emitted from two points and observe how the charges diffuse. In Fig. 2(d′), we find that the charges diffuse widely into the downstream region. In Fig. 2(e′), the charges do not diffuse much, but they flow within a certain width. In Fig. 2(f′), not only are the charges nondiffusing, but the flows tend to aggregate, creating a single flow.

In Euclidian space, the steady-state solution changes dramatically at *γ*_*C*_ = 0.5. The changes are deeply related to the diffusiveness of the transport and can be explained as follows. Assuming the lattice spacing *dx* and time scale *dt*, we can discretize the master equation of the transport.

3 $$\begin{array}{rcl}Q(x,t+dt) & = & \frac{{Q}^{\gamma }(x,t)}{{Q}^{\gamma }(x-2dx,t)+{Q}^{\gamma }(x,t)}Q(x-dx,t)\\  &  & +\frac{{Q}^{\gamma }(x,t)}{{Q}^{\gamma }(x+2dx,t)+{Q}^{\gamma }(x,t)}Q(x+dx,t)\\  &  & -\nu dtQ(x,t)+1dt\end{array}$$

where *Q*(*x*, *t*) is the density of the charges at time *t* and position *x*. The first and second terms denote the amount of current from the neighbors, *x* − *dx* and *x* + *dx*, respectively. The third and fourth terms denote the amount of dissipation and injection within the duration *dt*. For sufficiently small *dt* and *dx*, the ring network is equivalent to one-dimensional (1D) space. Assuming a uniform solution *Q*(*x*, *t*) = 1/*ν*, we can approximate the perturbation $\tilde{Q}(x,t)$ ignoring the terms of order greater than two on the right-hand side of Eq. (3). The following diffusion equation is derived.

4 $$\frac{\partial Q(x,t)}{\partial t}=D(\gamma )\frac{{\partial }^{2}Q(x,t)}{\partial {x}^{2}}-\nu Q(x,t)+1$$

5 $$D(\gamma )=\frac{1-2\gamma }{2}\frac{{(\delta x)}^{2}}{\delta t}$$

where *D*(*γ*) is the diffusion coefficient. The explicit form of *Q*(*x*, *t*) is given analytically as follows for *D*(*γ*) > 0.

6 $$Q(x,t)=\frac{1}{\nu }+\frac{1}{2\sqrt{\pi D(\gamma )t}}{e}^{-(\nu t+\frac{{x}^{2}}{4D(\gamma )t})}$$

It is straightforward to understand the sign of the diffusion coefficient, which is positive for *γ* < 1/2 and negative for *γ* > 1/2, implying that the diffusion–localization transition occurs at *γ* = 1/2. In the phase of the negative coefficient, currents appear to localize, and the steady-state configuration is localized depending on the initial conditions. The dimension of the Euclidian space generally does not change the diffusion coefficient. This is consistent with the numerical observation for the 2D Euclidian space in Fig. 2.

## Diffusion-localization transition on a typical network

These dramatic changes in the steady-state solution of Eq. (2) can be understood by considering the simplest case of 1D transport. In a lattice system, the nonlinear gravity flow is reduced to the normal diffusion equation by the continuum approximation. The order parameter, the diffusion coefficient, is explicitly evaluated; however, it is not available in a general network.

Focusing on the initial condition dependency for *γ* > *γ*_*C*_, we can examine the steady states of Eq. (2) and the stability of the solution.

Suppose that the transport equation, Eq. (2), has a steady-state solution ${Q}_{M}^{\ast }$, and we consider a small perturbation ${\tilde{Q}}_{M}(t)$ to this solution. Thus, the equation is linearized around the steady-state solution. The *i*-th node’s perturbation ${\tilde{Q}}_{i}(t)$ to a neighboring *j*-th node ${\tilde{Q}}_{j}(t)$ is described by a Jacobian matrix *J* as follows.

7 $${J}_{ij}=\{\begin{array}{ll}\gamma \sum _{k=1}^{N}{B}_{ki}(1-{B}_{ki})\frac{{Q}_{k}^{\ast }}{{Q}_{i}^{\ast }}-(1+\nu ) & (i=j)\\ {B}_{ij}-\gamma \sum _{k=1}^{N}{B}_{ki}{B}_{kj}\frac{{Q}_{k}^{\ast }}{{Q}_{i}^{\ast }} & (i\ne j)\end{array}$$

where ${B}_{ij}={A}_{ij}{Q}_{j}^{\ast \gamma }/{\sum }_{k}{A}_{ik}{Q}_{k}^{\ast \gamma }$. The value of *γ*_*C*_ at which the maximum eigenvalue of this Jacobian is zero determines the bifurcation point, which we call the transition point.

When the steady solution ${Q}_{M}^{\ast }$ is derived theoretically, we can calculate the eigenvalue using Eq. (7) easily. The examples refer to the complete network, the star network, the ring network with bilateral links, and the branch network comprising one root node and two leaf nodes controlled by injection parameters *F*_*root*_ and *F*_*leaf*_, respectively. For example, in the case of the complete network and the ring network, due to the symmetry of the topology, the stable steady-state solution is trivial, that is namely uniform, ${Q}_{M}^{\ast }=F/\nu$. Also, the star network has only two values of the steady state solution: one from the hub node, the other from the perimeters, ${Q}_{hub}^{\ast }=(N+\nu )F/\nu (1+\nu )$ and ${Q}_{peri}^{\ast }=F/(1+\nu )$, respectively. Equation (7) assigned with these steady solutions ${Q}_{M}^{\ast }$ easily derives the Jacobian matrix which is categorized into the circulant matrix and the eigen values. The branch network has more control parameters *F*_*root*_ and *F*_*leaf*_. The value of the root node converged to ${Q}_{root}^{\ast }={F}_{root}/(1+\nu )$ and the leaf node to ${Q}_{leaf}^{\ast }=(2(1+\nu ){F}_{leaf}+{F}_{root})/(2\nu (1+\nu ))$. This stability system is described by 3 × 3 Jacobian matrix as well. We list the transition points for typical network structures in Table 1. As shown in Table 1, the infimum values of the transition points *γ*_*C*_ take different values depending on the network structure in the limit *ν* = 0 and *N* = ∞.

**Table 1:** The transition points for typical networks.

Network topology	*γ*_*C*_(*N*, *ν*)	*γ*_*C*_(∞, 0)
Complete	$\frac{N-1}{N-2}(1+(1-\frac{1}{N})\nu )$	1
Star	$\frac{(N-1){(1+\nu )}^{2}+(1+\nu )}{(N-1)+(1+\nu )}$	1
Ring (*N* → ∞)	$\frac{1}{2}(1+\nu +\sqrt{{(1+\nu )}^{2}-1})$	1/2
Branch	1 + 2(1 + *ν*)*F*_*leaf*_/*F*_*root*_	1

## Real transport on a real network

**Figure 3:**
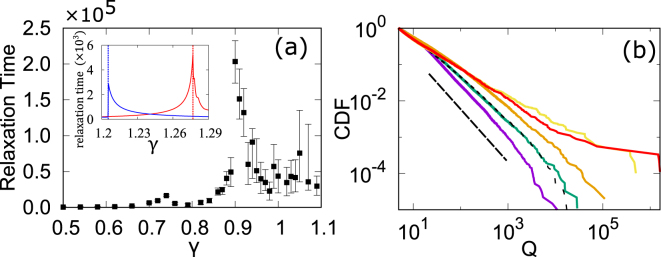
Basic properties of the gravity model for real interfirm trading network with approximately 0.6 million nodes. (**a**) Relaxation time as a function of *γ* for real interfirm trading network. The result of 500 samples with a randomly assigned initial state is plotted as the median, 25%, and 75% in each range. In the inset, the same plots for the complete network, (*N*, *ν*) = (10, 0.15), and the star network, (*N*, *ν*) = (50, 0.1), are displayed in red and blue, respectively; they agree with the theoretical values shown in Table 1. (**b**) Cumulative distributions of the steady-state solutions of Eq. (2) with *ν* = 0.1 normalized to 1 at *Q* = 5 for *γ* = 0 (blue), *γ* = 0.33 (green), *γ* = 0.6 (orange), *γ* = 1.0 (yellow), and *γ* = 1.1 (red). The dotted line denotes the sales distribution. The slope of the line is −1.4.

It is very interesting to estimate the transition point of *γ* for any given network structure. For this purpose, we introduce a numerical method based on observation of the relaxation times, as we can expect that a numerical solution will take a long time to converge to the steady solution if the control parameter is close to the transition point. Starting with a randomly assigned initial state, we numerically calculate the time evolution using Eq. (2), and at each time step we observe the change in each node, |*Q*_*i*_(*t* + 1) − *Q*_*i*_(*t*)|. If the quantity of nodes is greater than 10^−12^, we proceed with the time evolution; if it is less, we stop the iteration, and the relaxation time is defined by this time step. In Fig. 3(a), the relaxation time is plotted. Red and blue lines in the inset in Fig. 3(a) show the two typical cases that are analytically solvable, and we find that the relaxation time tends to diverge at a point that is very close to the theoretical transition point, as expected, in both cases.

Now, we investigate the properties of the generalized gravity interaction model when it is applied to an interfirm business relation network structure in the real world, as defined by the direction of money flow, for a network consisting of 627, 262 nodes and 3, 844, 684 directed links. The business relationship network is characterized by a power-law link-number distribution, which categorizes the network as a typical scale-free network with the power exponent 1.3~1.4^9,25^. It is also reported that the distribution of the sales, the charge distribution, obeys a power-law distribution with the exponent 1.0 as well^9,25^. Google’s PageRank system is known to follow a power-law distribution with an exponent that is almost identical to that for link numbers^16,18,26^.

We can estimate the transition point *γ*_*C*_ for this network by observing the relaxation time. The relaxation time is plotted as a function of *γ* in Fig. 3(a) and tends to diverge at *γ*_*C*_ = 0.9. It is also confirmed that for *γ* > *γ*_*C*_, there are multiple steady-state solutions, whereas for *γ* < *γ*_*C*_, the steady-state solution seems to be unique.

The value of *γ* for real business firms is estimated by the microscopic fitting of annual data on money flows between firms, using the model of the generalized gravity interaction with *γ*_*real*_ = *β*/*α* = 0.3/0.9~0.33^9^.

Figure 3(b) shows that if *γ* is zero, the transport is equivalent to normal diffusion on the network as we model it. Further, for *γ* < *γ*_*C*_, the power exponent of the distribution gradually changes. On the other hand, for transport with *γ* > *γ*_*C*_ = 0.9, which is in the localization state, the value of *γ* does not seem to affect the power exponent, and an extremely large node attracting current from the entire network emerges, as well as broad tails. In the real case, fitting the real sales value by the relation to the model, $\mathrm{log}\,{Q}_{M}=\alpha \,\mathrm{log}\,{S}_{M}-\,\mathrm{log}\,F$, we obtain the remaining two coefficients defined in our model: *F* = 95 and *α* = 0.89. The distribution of steady-state solutions ${Q}_{i}^{\ast }$ with these parameters are plotted in Fig. 3(b). The distribution of the real sales is transformed to *Q*_*i*_ by ${S}_{i}^{\ast }={(F{Q}_{i}^{\ast })}^{1/\alpha }$ with fitted values of *F* = 95 and *α* = 0.89, which obey a power law similar to that for our case with *γ* = 0.33. The model with the parameters obtained by microscopic fitting agreed well with a real sales distribution, whereas the degree distribution’s exponent, 1.4, and the sales distribution’s exponent, 1.0, are nontrivially related. It is suggested that the original form of the gravity law helps explain real money transport from the network structure.

## Discussion

In this paper, we investigated the general nonlinear transport of a scalar quantity in a complex network, which includes thermal transport in the special case where *γ* = 0. We found that there are two phases. The first is the diffusion phase for *γ* < *γ*_*C*_, in which charges diffuse over the entire system, as in thermal diffusion, and there is only one steady-state solution in the presence of dissipation and injection. The second phase is the localization phase with stronger nonlinearity, *γ* > *γ*_*C*_, in which charges flow preferentially to nodes with higher charge, resulting in irreversible river-like confluence patterns. In the latter phase, there are a small number of exceptional nodes that appear naturally and have very large charges that attract a huge amount of flow from the entire network. This may correspond to an oligopoly in the case of money flow. We can extract a basin structure that nearly fits the loopless tree-like structure from any given flow network by considering the limit of *γ* → ∞; for money flow among firms, the tree-like structure roughly represents the hierarchical relations of the production process of final goods from raw materials. The idea is almost equivalent to the basin structure of the flow network that we proposed previously^24^.

Finally, we also found that the network topology affects the transition point. It is numerically confirmed that the transition point, which can be estimated from the divergence of the relaxation time, of a real interfirm network is approximately 0.9, whereas the observed *γ* value is 0.33. According to our calculation, our gravity model is expected to be an empirical model for real money transport, and we can conclude that real money flow is in the diffusion phase state.

Important objectives of future work include clarifying the relationship between the transition point *γ*_*C*_ and the network structures in more detail, as well as investigating why *γ*_*C*_ differs from that in typical cases. Because transport is one of the most important aspects of network growth, criticality evaluating general transport based on the generalized gravity model is a promising area of research. There are many practical applications of the gravity interaction model as a model for money transport among firms; we can quantitatively estimate changes in money flow by changing the network structure. We expect that our model can provide the basic tools for simulation in economic risk evaluation.

## References

[1] Piermartini, R. & Rousova, L. Liberalization of air transport services and passenger traffic. *WTO Staff Working Paper* **ERSD-2008-06** (2008).

[2] Card D (2001). Immigrant inflows, native outflows, and the local labor market impacts of higher immigration. Journal of Labor Economics.

[3] Simini F, González M, Maritan A, Barabási A-L (2012). A universal model for mobility and migration patterns. Nature.

[4] Balcan D (2009). Multiscale mobility networks and the spatial spreading of infectious diseases. Proc. Natl. Acad. Sci. (USA).

[5] Viboud C (2006). Synchrony, waves, and spatial hierarchies in the spread of influenza. Science.

[6] Jung W, Wang F, Stanley H (2008). Gravity model in the korean highway. Europhys. Lett..

[7] Zipf GK (1946). The p1 p2/d hypothesis: On the intercity movement of persons. American Sociological Review.

[8] Tinbergen, J. *Shaping the World Economy; Suggestions for an International Economic Policy* (1962).

[9] Tamura K (2012). Estimation of flux between interacting nodes on huge inter-firm networks. Int. J. Mod. Phys. Conf. Ser..

[10] Caldarelli G (2012). A network analysis of countries’ export flows: firm grounds for the building blocks of the economy. PLoS ONE.

[11] Anderson J (1979). A theoretical foundation for the gravity equation. American Economic Review.

[12] Feenstra, R. Advanced international trade: Theory and evidence. *Princeton Univ. Press* (2003).

[13] Kaluza P, Koelzsch A, Gastner M, Blasius B (2010). The complex network of global cargo ship movements. J. R. Soc. Interface.

[14] Krings, G., Calabrese, F., Ratti, C. & Blondel, V. Urban gravity: a model for inter-city telecommunication flows. *J. Stat. Mech 2009* **L07003** (2009).

[15] Brin S, Page L (1998). The anatomy of a large-scale hypertextual web search engine. Computer Networks and ISDN Systems.

[16] Fronczak A, Fronczak P (2009). Biased random walks on complex networks: the role of local navigation rules. Phys. Rev. E.

[17] Baronchelli A, Pastor-Satorras R (2010). Mean-field diffusive dynamics on weighted networks. Phys. Rev. E.

[18] Noh JD, Rieger H (2004). Random walks on complex networks. Phys. Rev. Lett..

[19] Tadić B, Rodgers GJ, Thurner S (2007). Transport on complex networks: Flow, jamming and optimization. International Journal of Bifurcation and Chaos.

[20] Watanabe H, Takayasu H, Takayasu M (2012). Biased diffusion on japanese inter-firm trading network: Estimation of sales from network structure. New J.Phys..

[21] Kleinberg J (1999). Authoritative sources in hyperlinked environment. J. ACM.

[22] Sood V, Grassberger P (2007). Localization transition of biased random walks on random networks. Phys. Rev. Lett..

[23] Fronczak A, Fronczak P (2006). Networks with given two-point correlations: hidden correlations from degree correlations. Phys.Rev. E.

[24] Tamura K, Takayasu H, Takayasu M (2015). Extraction of conjugate main-stream structures from a complex network flow. Physical Review E.

[25] Takayasu, M. *et al*. Massive economics data analysis by econophysics methods - the case of companies’ network structure. *Annual Report of the Earth Simulator Center April 2007*–*March 2008* **263** (2008).

[26] Ohnishi T, Takayasu H, Takayasu M (2009). Hubs and authorities on japanese inter-firm network: Characterization of nodes in very large directed networks. Progress of Theoretical Physics Supplement.

